# Ferroptosis: Regulatory mechanisms and potential targets for bone metabolism: A review

**DOI:** 10.1097/MD.0000000000039158

**Published:** 2024-09-27

**Authors:** Yongjie Zhang, Kangyi Hu, Zhengya Shang, Xiaorui Yang, Linzhong Cao

**Affiliations:** a Gansu University of Traditional Chinese Medicine, Lanzhou, China; b The Affiliated Hospital of Gansu University of Chinese Medicine, Lanzhou, China.

**Keywords:** bone homeostasis, ferroptosis, iron, organelles, signal pathway

## Abstract

Bone homeostasis is a homeostasis process constructed by osteoblast bone formation and osteoclast bone resorption. Bone homeostasis imbalance and dysfunction are the basis for the development of various orthopedic diseases such as osteoporosis, osteoarthritis, and steroid-induced avascular necrosis of femoral head. Previous studies have demonstrated that ferroptosis can induce lipid peroxidation through the generation of reactive oxygen species, activate a number of signaling pathways, and participate in the regulation of osteoblast bone formation and osteoclast bone resorption, resulting in bone homeostasis imbalance, which is an important factor in the pathogenesis of many orthopedic diseases, but the mechanism of ferroptosis is still unknown. In recent years, it has been found that, in addition to iron metabolism and intracellular antioxidant system imbalance, organelle dysfunction is also a key factor affecting ferroptosis. This paper takes this as the starting point, reviews the latest literature reports at home and abroad, elaborates the pathogenesis and regulatory pathways of ferroptosis and the relationship between ferroptosis and various organelles, and summarizes the mechanism by which ferroptosis mediates bone homeostasis imbalance, with the aim of providing new directions for the research related to ferroptosis and new ideas for the prevention and treatment of bone and joint diseases.

## 1. Introduction

In 2003, Sonam Dolma et al found that rubber has a selective lethal effect on cancer cells, but the cell death mode is in a more specific way, without nuclear morphological changes or DNA breaks.^[1]^ Yang et al^[2]^ further found that this pattern of cell death is iron-dependent and can be inhibited by iron chelators. In 2012, Professor Dixon, a chemical biologist at Columbia University in the United States, first proposed the concept and pointed out that ferroptosis is a novel and unique form of programmed cell death and that disorders of iron metabolism, lipid peroxidation, and glutathione (GSH) are the characteristics of ferroptosis.^[3]^ With the deepening of research, scholars have made further discoveries about ferroptosis, which is different from necrosis, apoptosis, and autophagy in cell morphology, and does not have typical necrotic morphological characteristics, mainly manifested as mitochondrial contraction, increased membrane density, decreased or disappeared mitochondrial crest, intracellular GSH depletion, decreased glutathione peroxidase 4 (GPX4) activity, etc.^[4]^ Since then, ferroptosis has been found to be closely associated with the pathophysiological processes of many diseases, such as neurological diseases,^[5]^ cardiovascular diseases,^[6]^ liver diseases,^[7]^ lung diseases,^[8]^ kidney diseases,^[9]^ infectious immunity,^[10]^ and cancer.^[11]^

Bone homeostasis is a dynamic equilibrium process constructed by osteoblast bone formation and osteoclastic bone resorption. The maintenance of bone homeostasis is affected by many factors, and bone homeostasis imbalance affects the occurrence and development of a variety of bone and joint disorders, such as osteoporosis, osteoarthritis, and steroid-induced avascular necrosis of femoral head (SANFH). With the deepening of research on bone metabolism, a large number of studies at home and abroad have confirmed the close relationship between ferroptosis and bone homeostasis.^[12]^ Iron is an essential trace element that can synthesize substances such as hemoglobin and Fe–S atomic clusters and participate in a variety of physiological processes, such as cell proliferation, metabolic reactions, and immune defense. However, iron accumulation can affect the activity and function of bone marrow mesenchymal stem cells (BMSCs), osteoblasts (OB), and osteoclasts (OC) by increasing intracellular reactive oxygen species (ROS) levels and lipid peroxidation, and even induce cellular ferroptosis and disrupt bone homeostasis. However, there is little literature that can comprehensively and systematically elucidate the occurrence and regulation mechanisms of ferroptosis and fails to comprehensively summarize the relationship between ferroptosis and organelles and the impact of ferroptosis on bone homeostasis. In view of this, we reviewed the occurrence and regulation mechanism of cellular ferroptosis and its impact on bone homeostasis and elaborated and summarized the relationship between organelle dysfunction (including mitochondria, endoplasmic reticulum, Golgi apparatus, and lysosome) and ferroptosis at home and abroad in recent years, focusing on further understanding the pathogenesis of ferroptosis and new therapeutic targets for osteoarticular diseases.

## 2. Overview of iron metabolism

**Figure 1.**
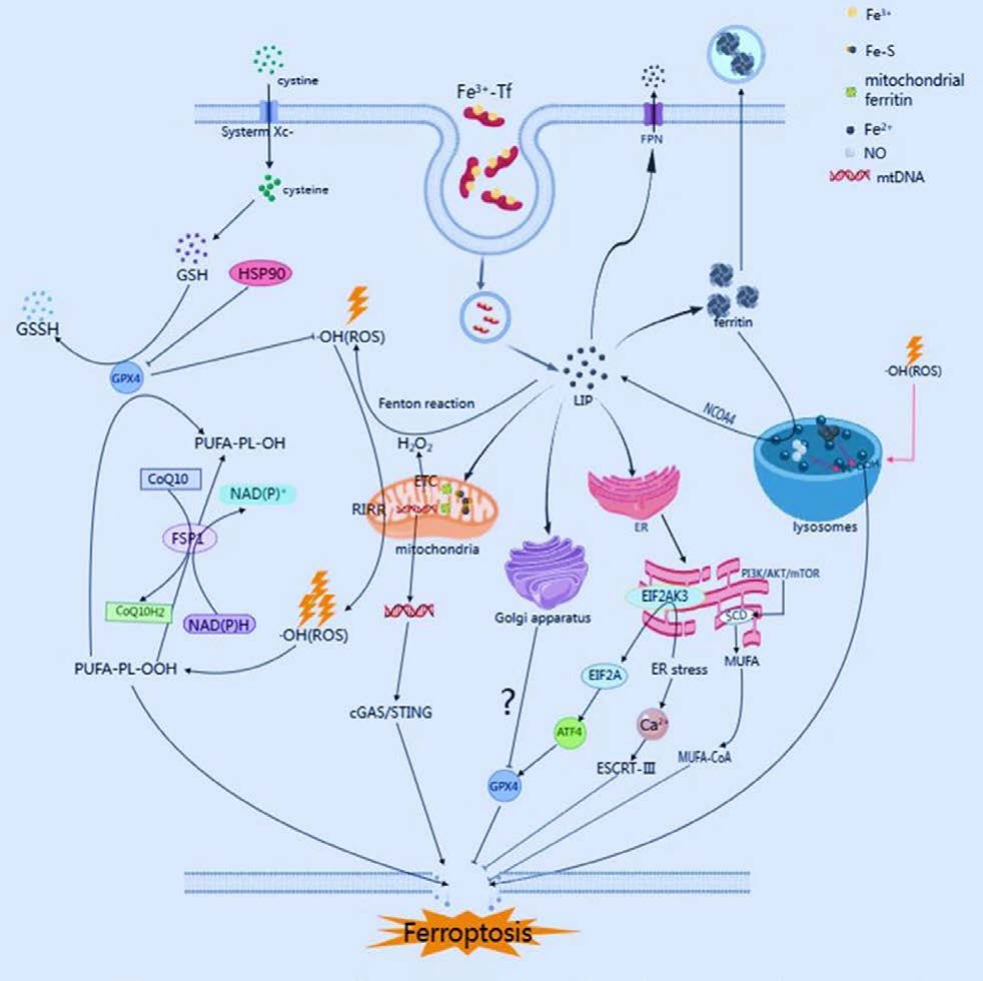
Mechanism of ferroptosis: effects of iron metabolism, antioxidant system, cell membrane repair system, and organelles on ferroptosis.

Iron, as a trace element, is necessary for the normal physiological activity of cells. It is involved in many necessary physiological activities and is essential for the function and survival of cells.^[13]^ It is mainly derived from senescent erythrocytes and diet. Food contains trivalent nonheme iron and divalent heme iron, of which Fe^3+^ is reduced to Fe^2+^ by Dcytb iron reductase, transferred to the cytoplasm of intestinal epithelial cells by direct uptake by divalent metal transporters on the intestinal epithelial cell membrane, and then transported from intestinal epithelial cells to plasma through iron transporters. Transferrin (Tf) in plasma binds to Fe^3+^ to become Fe^3+^-Tf, which enters the cell as endosomes with the help of a cell surface iron transporter receptor. After entering the cell, the proton pump on the endosomal membrane carries out ion exchange, and the pH value in the membrane decreases, resulting in the release and decomposition of Fe^3+^-Tf in the endosome, which is reduced to Fe^2+^ under the catalysis of iron reductase, which binds to ferritin and forms a variety of enzymes in the cytoplasm, such as: lipoxidase, nicotinamide adenine dinucleotide dehydrogenase, catalase, peroxidase, etc, and enters the mitochondria to synthesize Fe–S clusters, heme and mitochondrial ferritin, participating in the tricarboxylic acid cycle and aerobic respiration.^[14]^ Under normal conditions, iron metabolism is tightly controlled and iron uptake and output are in a state of dynamic equilibrium.^[15]^ As shown in Figure 1.

## 3. Ferroptosis regulation mechanism

Iron accumulation and lipid peroxidation are the main characteristics of ferroptosis, and ROS and lipid peroxides are the “executors” of cellular ferroptosis. When the body or cell iron metabolism is abnormal and the iron ions in the cell continue to accumulate, a large amount of ROS will be generated, and the polyunsaturated acid side chain associated with the ROS biofilm and macromolecules (such as nucleic acids) undergo lipid peroxidation reactions, forming lipid peroxides such as malondialdehyde and 4-hydroxynonenal, leading to structural and functional changes in the cells and even induced cell death. Iron metabolism disorders and antioxidant system imbalances are important factors in the occurrence of iron and play an important role in the occurrence of cellular ferroptosis.^[16,17]^ As shown in Figure 1.

### 3.1. Regulation of iron metabolism

#### 3.1.1. Iron absorption pathway

Iron metabolism in the body and in cells is affected by many factors. The body’s iron metabolism is mainly regulated by hepcidin, which is a hormone-like polypeptide secreted by the liver, and its formation is mainly regulated by bone morphogenetic protein/SMAD and signaling converters and transcriptional activator 3 and other signaling pathways, bone morphogenetic protein on the cell surface through the recruitment of hepcidin regulatory protein, so that SMAD phosphorylation thereby activating hepcidin-related gene transcription.^[18]^ After hepcidin is secreted by liver cells, it is released into the circulatory system, which combines with the small intestinal membrane iron transport protein (FPN) to form the hepcidin–FPN complex, induces FPN internalization and degradation, inhibits the absorption of iron by the intestinal epithelium, and reduces the content of iron ions in the blood circulation, thereby achieving the purpose of regulating iron metabolism.^[19]^ The intracellular iron metabolic balance is primarily regulated by the iron regulatory protein/iron responsive element (IRP/IRE).^[20–22]^ IRPs include IRP1, an RNA-binding protein found within the cytoplasm of cells and encoded by the Irp2 gene. The function and structure of IRP2 and IRP1 are similar, both of which regulate intracellular iron metabolism, but IRP2’s ability to regulate iron metabolism is more prominent than that of IRP1.^[23]^ IRE is a stemloop structure nucleotide sequence consisting of a six-base loop (5′-CAGUGX-3′ ring) located at the end of the RNA helix containing a protruding nucleotide region inside, which is key for IRPs to recognize IREs.^[24–26]^ When cellular iron is deficient, IRPs bind to IREs in the 5′-untranslated region (5′-UTR) on ferritin mRNA, inhibiting ferritin mRNA translation to reduce iron storage. At the same time, IRPs bind to the IRE of the 3′-UTR of transferrin receptor mRNA to increase iron uptake and utilization. When iron content is high, IRRP binding to the IRE is inhibited, promoting transferrin receptor mRNA degradation, enhancing the transcription of membrane transferrin and ferritin, and promoting efflux and storage of iron ions to reduce intracellular iron levels.^[27,28]^

#### 3.1.2. Ferritin autophagy pathway

Ferritin, as the main form of iron storage, consists of heavy chains and light chains, of which the heavy chains contain ferrous oxidase, which can oxidize Fe^2+^ to stable Fe^3+^ for storage, and the light chains can directly chelate Fe^2+^. Ferritin synthesis and autophagy are important ways for cells to regulate iron ion metabolism and play a vital role in maintaining cellular physiological iron homeostasis, which is mainly regulated by nuclear receptor coactivator 4 (NCOA4). As a key regulator of ferritin autophagy, NCOA4’s C-terminal domain contains a site that binds to ferritin heavy chains, which can recognize and target binding to the R23 domain of ferritin heavy chains and interact with autophagy-related factors ATG and LC3 to mediate the recognition of ferritin by autophagies and transport them to lysosomes to degrade and release free iron ions, and its mediated ferritin autophagy constitutes an important part of intracellular iron metabolism.^[29,30]^ However, excessive autophagy of ferritin leads to a large accumulation of intracellular iron ions, which promotes iron accumulation in cells.^[31]^ Gryzik M et al^[32]^ found that erasin, as an inducer of ferroptosis, leads to ferritin degradation and iron release through NCOA4-mediated ferritin autophagy and elevated intracellular free iron levels, thereby promoting ferroptosis in cells. Yu F et al^[33]^ found that by inhibiting O-(linkage)-N-acetylglucosamine glycosylation modification, it can promote ferritin autophagy and ferritin heavy chain degradation, resulting in cellular ferroptosis. The above studies show that ferritin autophagy plays a key role in the regulation of iron metabolism and is a key mediator of cellular ferroptosis caused by iron accumulation.

#### 3.1.3. Extracellular vesicles pathway

Extracellular vesicles are membrane-like structures released into the outside of cells after fusion of intracellular vesicles with cell membranes. They can carry proteins, mRNA, lncRNA, and miRNA, and play a role in regulating intercellular signaling, immune response, homeostasis, and cell physiology and pathology.^[34]^ Extracellular vesicles are closely related to cellular ferroptosis and can inhibit cellular ferroptosis by regulating intracellular ferritin and iron ion content, lipid metabolism, and amino acid metabolism. Their related research has attracted extensive attention from scholars. Extracellular vesicles-mediated efflux of ferritin is one of the main forms of regulating intracellular iron content, and Prominin2 is an iron-dead stress response protein that is able to produce ferritin-containing extracellular vesicles, promote extracellular transport of iron, inhibit cellular ferroptosis, and block extracellular vesicles-mediated ferritin output, which can lead to continuous accumulation of intracellular iron.^[35]^ Yang RZ et al^[36]^ found that extracellular vesicles were able to reverse glucocorticoid-induced osteogenesis inhibition and prevent glucocorticoid-induced osteoporosis, which is achieved by exporting ferritin and inhibiting ferritin’s autophagy-dependent ferroptosis. Lipid peroxide accumulation is the main factor and characteristic of ferroptosis, and the regulation of intracellular lipid metabolism by extracellular vesicles is another key mechanism affecting ferroptosis. Zhang H et al^[37]^ found that after the secretion of extracellular vesicles miR-522, it can inhibit the expression of lipoxygenase 15 arachidonate and reduce the level of lipid ROS, whereas blocking the secretion of extracellular vesicles miR-522 raises the level of ferroptosis, demonstrating that extracellular vesicles are associated with ferroptosis lipid peroxidation. In addition, extracellular vesicles are also able to regulate cellular ferroptosis by regulating amino acid metabolism. Lin F et al^[38]^ showed in vivo and in vitro experiments that ferroptosis is a key factor in carbon tetrachloride-induced liver injury in mice, while extracellular vesicles can significantly attenuate lipid peroxidation caused by ubiquitination of carbon tetrachloride and inhibit cellular ferroptosis by maintaining SLC7A11 function. Yuan Y et al^[39]^ also found that extracellular vesicles miR-22-3P reduces cellular ferroptosis by targeting ASCL4, a key regulatory molecule of ferroptosis. In conclusion, extracellular vesicles are closely related to cytosis and will become one of the important targets for regulating cellular ferroptosis and preventing and treating clinical diseases. However, the specific mechanism between extracellular vesicles and ferroptosis is unclear and needs to be further studied.

### 3.2. Antioxidant system regulation

#### 3.2.1. System Xc‐/GSH/GPX4 axis

System Xc‐/GSH/GPX4 axis imbalance is one of the main causes of cell ferroptosis. If GPX4 or System Xc‐ are inhibited, intracellular ROS and lipid peroxides will continue to accumulate, inducing cell ferroptosis. System Xc‐, as a widely distributed transporter protein in the phospholipid bilayer, is an important component of the intracellular antioxidant system, consisting of SLC3A2 and SLC7A11, capable of transporting cystine outside the cell to further synthesize GSH. GSH, as a “gatekeeper” in the process of ferroptosis, exists in cells in the form of GSH and oxidized glutathione, playing a vital antioxidant role in cellular oxidative stress. The decrease in SLC7A11 expression level inhibits cystine uptake by System Xc‐, reduces intracellular GSH content, reduces GPX4 activity and cellular antioxidant capacity, increases intracellular ROS, and leads to cellular ferroptosis. Song X et al^[40]^ found that selective inhibition of System Xc‐ can significantly reduce the level of GSH in cells, exacerbate the accumulation of ROS, and mediate the occurrence of ferroptosis. Liu M et al^[41]^ believed that SLC7A11-mediated cystine uptake plays a necessary role in the inhibition of lipid peroxidation and cellular ferroptosis and found that NRF2 and activating transcription factor-4 (ATF4) can activate SLC7A11, and SLC7A11 can promote GPX4 protein synthesis by activating the mTORC1-4EBP, while P53, BAP1, ATF3, and BECN1 can inhibit SLC7A11 function. Shi J et al^[42]^ found that homocysteine can inhibit the expression of SLC7A11 and GPX4 proteins, increase intracellular ROS levels, and be dose-dependent, confirming that the System Xc‐/GSH/GPX4 axis is important for regulating ferroptosis. In short, the System Xc‐/GSH/GPX4 axis, as an important pathway for regulating oxidative stress, is an important target for regulating cellular ferroptosis, and the combined regulation of the System Xc‐/GSH/GPX4 axis can more effectively prevent and control ferroptosis-related diseases.

#### 3.2.2. FSP1/NAD(P)H/CoQ10 axis

The ferroptosis inhibitory protein 1 (FSP1)/NAD(P)H/Coenzyme Q10 (CoQ10) axis is another key pathway that regulates ferroptosis, and loss of FSP1 can also lead to phospholipid peroxidation at certain GPX4 expression levels.^[43,44]^ The FSP1 gene is located on human chromosome 10q22.1. FSP1 is structurally similar to human caspase-independent proapoptotic proteins and is also known as a mitochondria-associated apoptosis-inducing factor.^[45]^ Its structures mainly include the reduced nicotinamide adenine dinucleotide oxidoreductase domain that can catalyze cytochrome c and reduce other electron receptors, domains that bind nonspecifically to DNA, and domains that bind to the cofactor flavin adenine dinucleotide. Recent studies have found that FSP1 not only participates in the regulation of apoptosis but also has an important inhibitory effect on the process of ferroptosis.^[46]^ As an oxidoreductase, FSP1 can reduce ubiquinone on cell membranes to ubiquinol through NAD(P)H, which directly reduces lipid free radicals to terminate lipid peroxidation and indirectly regenerates oxidized α-tocopherol free radicals, thereby acting as antioxidants. CoQ10 is a fat-soluble quinone compound that often exists in 3 different redox states, including CoQ, CoQH, and CoQH2, and it is the only lipid antioxidant that can be self-synthesized in vivo. CoQH2 is a lipophilic radical-trapping antioxidant with a strong antioxidant effect that can reduce intracellular ROS and lipid free radicals, thus inhibiting ferroptosis. In addition, FSP1 can also inhibit ferroptosis through an endosomal sorting complex repaired for transport (ESCRT-III)-dependent membrane requirement mechanism, but the specific regulatory mechanism remains unclear.^[47]^ In summary, the FSP1/NAD(P)H/CoQ10 axis is another important pathway for regulating intracellular oxidative stress, which plays an irreplaceable role in the process of cellular ferroptosis and provides more targets for inhibiting oxidative stress and intervening in the study of cellular ferroptosis.

### 3.3. ESCRT-III-dependent membrane repair pathway

The importance of lipid peroxidation in the process of cellular ferroptosis is well understood, but its mediated membrane damage as a key cause of cytosis is poorly understood. ESCRT-III is a “tinkerer” in the repair process of cell membrane damage, and ESCRT-III-dependent membrane repair pathways can inhibit the onset of ferroptosis in some cases.^[48]^ The ESCRT system is able to recognize ubiquitinylated modified membrane proteins and mediate endocytic vesicular budding and polyvesicular body formation, participating in processes such as cytoplasmic division and autophagy. In 2014, Jimenez et al demonstrated the important role of the ESCRT membrane repair system in the process of plasma membrane small wound repair.^[49]^ Subsequently, Scheffer et al^[50]^ verified the mechanism of the ESCRT membrane repair system in the rapid repair of large plasma membrane wounds. In recent years, it has been found that ESCRT-III is composed of 7 subunits, and the interaction between the subunits forms a spiral filamentous structure. ESCRT-III plays a central role in the membrane shearing process mediated by the ESCRT system.^[51,52]^ ESCRT-III repairs the “wound” on the cell membrane mainly through 2 ways, namely, budding and forming endosome formation, both of which can form extracellular vesicles, thereby promoting cell membrane repair and “wound” healing.^[49,53]^ Pedrera L et al found that the continuous increase of Ca^2+^ is a hallmark of membrane rupture in ferroptosis cells, and Ca^2+^ can induce the activation of the ESCRT III-dependent membrane repair system. They confirmed that the ESCRT-III-mediated membrane repair system can effectively inhibit cell ferroptosis by resisting membrane perforation and delaying cell “death.”^[54]^ The above studies confirm that cell membrane damage is an important influencing factor of ferroptosis, and it can effectively inhibit cellular ferroptosis by activating the cell membrane repair system, but there are few relevant studies in this regard, and the specific regulatory mechanism of cell membrane repair in the process of ferroptosis is not clear. It may become a research hotspot in regulating cellular ferroptosis in the future and an effective intervention target for ferroptosis-mediated related diseases. In summary, iron accumulation, lipid peroxidation, and cell membrane damage are indispensable in ferroptosis, which can be modulated by regulating iron metabolism, addressing oxidative stress, and promoting cell membrane repair.

## 4. Ferroptosis and organelles

Ferroptosis is a complex process, the mechanism of which has not yet been elucidated so far. Recent studies have found that ferroptosis is not only regulated by the above signaling pathways and targets but also closely related to organelles such as mitochondria, endoplasmic reticulum, and lysosomes,^[55]^ as shown in Figure 1.

### 4.1. Ferroptosis and endoplasmic reticulum

The endoplasmic reticulum is an essential organelle in eukaryotic cells, involved in intracellular biosynthesis, signaling, and coordination of intracellular protein folding, transport, and posttranslational modifications. The endoplasmic reticulum provides a suitable processing environment for new peptides, and the proteins targeting the endoplasmic reticulum are synthesized in the ribosomes attached to the endoplasmic reticulum membrane, guided into the endoplasmic reticulum membrane through the terminal, folded with a special three-dimensional shape as a template, and finally formed into secreted proteins and targeted secretion after glycogen and disulfide bond modification. Recent studies have found that there is a synergistic effect between ferroptosis and apoptosis, and endoplasmic reticulum stress is the “connection point” between the 2.^[56,57]^ Endoplasmic reticulum stress can promote the production of ROS, induce lipid peroxidation, and lead to cellular ferroptosis. Ferroptosis can also induce endoplasmic reticulum stress by inhibiting System Xc‐, and protein kinase R-like endoplasmic reticulum kinase (PERK)/ATF4/C/EBP homologous protein (CHOP), which is an important signaling pathway to regulate pathological reactions such as iron accumulation and endoplasmic reticulum stress. Chen et al^[58]^ found that silencing ATF4 by siRNA significantly reduced the expression level of System xc‐ and increased the sensitivity of human gliomas to erastin and the GPX4 inhibitor RSL3-induced ferroptosis. Xu M et al^[59]^ further found that ferroptosis promotes ulcerative colitis through endoplasmic reticulum stress-mediated death of intestinal epithelial cells and that GSK414, an inhibitor of PERK, can inhibit endoplasmic reticulum stress-mediated intestinal epithelial cell ferroptosis to alleviate ulcerative colitis. Subsequently, Ru Q et al^[60]^ found that ferroptosis is closely related to endoplasmic reticulum stress; iron accumulation can not only induce cellular ferroptosis through oxidative stress but also induce an unfolded protein response, followed by the activation of the PERK-eIF2α-ATF4-CHOP pathway mediated by endoplasmic reticulum stress; and p53-independent PUMA expression mediated by the CHOP signaling pathway, which participates in the synergy between ferroptosis and apoptosis. Another study^[61]^ has found that the PERK/eIF2α signaling pathway can influence the production of ROS, which in turn regulates the process of cellular ferroptosis. The above studies confirmed that the endoplasmic reticulum is related to ferroptosis and can be used as an important target for regulating ferroptosis and preventing and treating related diseases.

### 4.2. Ferroptosis and mitochondria

Mitochondria are the “power workshops” of cells and can provide energy support for the physiological activities of cells. At the same time, mitochondria are also the death regulatory centers of cells, which can regulate intracellular ROS levels and regulate the proliferation and death processes of cells^[62]^ Iron is one of the most abundant metals in mitochondria, and its involvement in the transmission of mitochondrial electron respiration chains is closely related to ATP synthesis. Abnormalities in mitochondrial function and structure are closely related to cellular ferroptosis.^[63,64]^ ROS accumulation in mitochondria, reduction or disappearance of the mitochondrial crest, changes in mitochondrial membrane potential, increased mitochondrial membrane density, and mitochondrial reduction are important features of cellular ferroptosis.^[65,66]^ On the one hand, mitochondrial DNA can be mutated, resulting in dysfunction of the mitochondrial respiratory chain and abnormal leakage of electrons, and free electrons react with oxygen molecules to produce superoxide anions. On the other hand, it can lead to mitochondrial membrane potential depolarization and voltage-dependent anion channel (VDAC) opening. VDAC is a multifunctional channel protein that can mediate intracellular mitochondrial outer membrane ion transport, and the opening of VDAC can promote the production of mitochondrial ROS. Wang H et al^[67]^ believe that iron accumulation can lead to mitochondrial membrane potential depolarization and VDAC opening, and VDAC opening can promote the production of mitochondrial ROS. In addition, after mitochondrial membrane depolarization, the potential stability of the mitochondrial membrane decreases, mitochondrial membrane permeability conversion pores open, and after ROS enters mitochondria, the ROS-induced ROS release mechanism is activated, forming a vicious circle and inducing an ROS outbreak, which in turn leads to ferroptosis in cells. Yang Y et al^[68]^ found that the ferroptosis inducer erastin, in addition to inhibiting the transporter SLC7A11, can also directly bind to VDAC2 to induce ferroptosis. In addition, mitochondrial ferritin (FtMt) is the main form of iron ions stored in the mitochondria of cells, which can maintain iron homeostasis in cells and mitochondria; overexpression of FtMt can reduce the damage caused by oxidative stress to cells and reduce osteoblast ferroptosis; and inhibition of FtMt can induce mitochondrial autophagy through the ROS/PINK1/Parkin pathway, resulting in increased osteoblast ferroptosis.^[69]^ In short, mitochondrial dysfunction is also a non-negligible factor in the occurrence of ferroptosis, and regulating mitochondrial function will become an important way to regulate ferroptosis.

### 4.3. Ferroptosis and other organelles

Lysosomes and Golgi apparatuses are also involved in the process of cellular ferroptosis. As a “digestive organ” in eukaryotic cells, lysosomes contain proteases and nucleases, which have the function of decomposing biological macromolecules such as proteins, polysaccharides, and nucleic acids. When external substances enter the cell, or the cell itself is abnormalized in the cytoplasm, and the cell is in senescence, the lysosomes in the cell rupture and release hydrolases, which in turn cause the cell to be digested, decomposed, and die. Lysosomal dysfunction is closely associated with iron accumulation-mediated cellular ferroptosis and acts as a “bridge” between autophagy and ferroptosis.^[70]^ Torii S et al^[71]^ found that lysosomal activity inhibitors can inhibit intracellular iron accumulation and reduce the accumulation of ferroptosis-related ROS by inhibiting intracellular transport of transferrin or autophagy degradation of ferritin, confirming that lysosomes are linked to cellular iron balance and ROS production. Gao H et al^[72]^ found that pharmacological blockade of histone activity or V-ATPase inhibited erastin-induced ferroptosis, validating the importance of lysosomes in the process of cellular ferroptosis. The Golgi apparatus is a membrane organelle within eukaryotic cells that processes, modifies, classifies, and packages proteins synthesized by the endoplasmic reticulum before transporting them to specific parts of the cell or secreting them outside the cell. Alborzinia H et al^[73]^ found that there is a certain association between Golgi dysfunction and cellular ferroptosis and that treatment with Golgi apparatus inhibitors (such as BFA, GCA, or AMF-26) can reduce intracellular GSH levels, lead to ROS accumulation, induce ferroptosis, and confirm the correlation between cellular ferroptosis and Golgi. The above studies show that the abnormal function of lysosomes and Golgi affects the occurrence and development of cellular ferroptosis, which can be used as an important target organelle for the regulation of ferroptosis, but there are fewer studies on the regulation of ferroptosis through lysosomes and Golgi apparatus, and the specific mechanism is still unclear, which is to be further studied.

## 5. Ferroptosis and bone homeostasis

**Figure 2.**
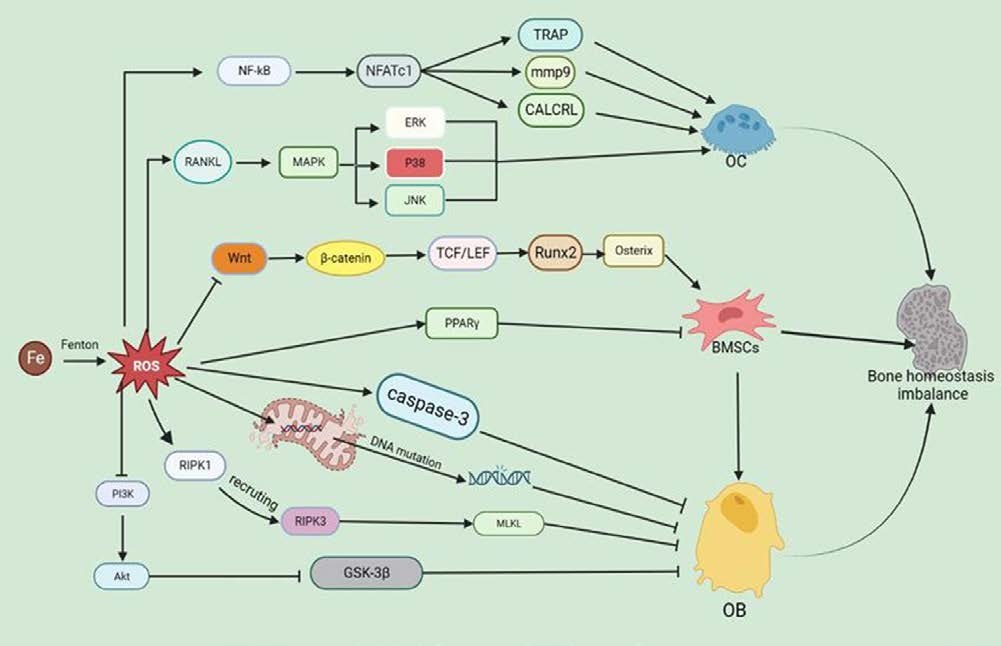
Ferroptosis and bone homeostasis: NF-kB signaling pathway; RANKL signaling pathway; Wnt signaling pathway; PPARγ signaling pathway; RIPK1/RIPK3/MLKL signaling pathway; PI3K/AKT/GSK-3β signaling pathway; DNA mutation. PPARγ = peroxisome proliferator-activated receptor γ, RANKL = receptor activator of nuclear factor-κB ligand.

Iron accumulation is an important influence on the imbalance of bone homeostasis in the human body, which is significantly negatively correlated with bone mass.^[74]^ BMSCs, OB, and OC are important target cells for regulating bone homeostasis. The increase in iron ion content can inhibit the activity of BMSCs, resulting in osteogenic-lipogenic differentiation disorders, reducing osteoblast bone formation ability, and promoting osteoclast proliferation, as shown in Figure 2.

### 5.1. Ferroptosis and BMSCs

Osteogenic and lipogenic differentiation of BMSCs maintains the balance of bone tissue and adipose tissue and plays a crucial role in bone reconstruction.^[75]^ The Wnt signaling pathway, peroxisome proliferator-activated receptor γ signaling pathway, and Runt-associated transcription factor 2 (Runx2) signaling pathway are important pathways for regulating osteogenic-lipogenic differentiation of BMSCs. Rozen^[76]^ found that after treatment with different concentrations of ammonium ferric citrate, the activity and osteogenic differentiation ability of BMSCs were inhibited, bone nodules and calcium salt deposition were reduced, and the content of ROS and lipid peroxides in BMSCs increased, all of which were dose-dependent. Further experiments found that iron accumulation was through the inhibition of the Wnt signaling pathway to reduce the osteogenic differentiation ability of BMSCs, and activation of the Wnt signaling pathway could reduce the ferroptosis of BMSCs and restore their osteogenic differentiation ability. Zhang et al^[77]^ found that radiation can reduce hepcidin activity, increase iron levels in the body, and inhibit the osteogenic differentiation ability of BMSCs by reducing Runx2. Han Yanjiu et al^[78]^ used different concentrations of ferrous citrate to intervene in BMSCs and found that iron accumulation could not inhibit the proliferation of human BMSCs, but iron accumulation could increase the level of ROS in human BMSCs, inhibit the expression of c-Maf and Runx2 in human BMSCs, promote the expression of peroxisome proliferator-activated receptor γ in human BMSCs, reduce the number of calcium nodules, and increase the formation of lipid droplets. The use of antioxidants to inhibit ROS can upregulate c-Maf. The expression and reversal of the effect of iron accumulation on osteogenic-lipogenic differentiation of human BMSCs. However, Yao X et al^[79]^ concluded that the experimental conclusions were slightly different from Han Yanjiu et al. Yao X et al treated BMSCs with ferric ammonium citrate and found that the expression of caspase-3 and BAX proteins was increased, the expression of Bcl-2 protein was reduced, and the activity of BMSCs was reduced, which confirmed that iron accumulation in BMSCs could not only lead to abnormal osteogenesis-osteogenic differentiation but also inhibit the activity and proliferation of BMSCs. In general, iron accumulation can affect the expression of the above multiple signaling pathways and related proteins, leading to osteogenic-lipogenic differentiation disorders of BMSCs and inhibiting the activity and proliferation ability of BMSCs, resulting in bone homeostasis imbalance.

### 5.2. Ferroptosis and OC

OC are differentiated from monocytes and macrophage precursor cells, and the receptor activator of nuclear factor-κB ligand (RANKL) is the main key molecules regulating the differentiation process of OC. OC have a bone resorption function that is dynamically balanced with osteoblast bone formation and plays a necessary part in the process of bone reconstruction. However, excessive differentiation of OC can lead to increased bone resorption, bone homeostasis imbalance, and participation in the development of bone and joint diseases. As one of the important factors that can affect the function of OC, if iron ions accumulate in OC, the level of intracellular ROS increases, activating the RANK/RANKL/OPG signaling pathway and upregulating the expression of RANKL. RANKL binds to RANK and activates multiple downstream signaling pathways, such as the mitogen-activated protein kinase (MAPK) and nuclear factor-κB pathways, promoting abnormal differentiation of OC, resulting in massive loss of bone mass.^[80–82]^ After Yan C et al intervened in bone tissue with the ferroptosis inducer Erastin, the cell membrane negative ion channel protein VDAC2/3 of OC was activated, resulting in a large number of iron ions being transported into the cell, generating a large amount of ROS, and inducing RANKL to stimulate the differentiation of monocyte macrophage lineages into OC.^[83]^ Li L^[84]^ and Park YR^[85]^ et al found that RANKL can induce continuous phosphorylation of ERK through the MAPK signaling pathway, increase the level of c-Fos protein, and enhance the expression of metalloproteinase 9, promoting osteoclast proliferation. Feng H et al^[86]^ believed that the MAPK signaling pathway was the main mechanism of osteoclast differentiation and experimentally found that the MAPK signaling pathway activated its downstream signaling molecule P38, and after P38 activation, it induced STAT1 phosphorylation at Ser727, promoting the expression and secretion of interferon γ-inducible factors, thereby stimulating osteoclast precursor cell differentiation and osteoclast adhesion. In summary, iron accumulation mainly affects the bone resorption function of OC through the RANK/RANKL/OPG signaling pathway, downstream MAPK signaling pathway, and NF-κB pathway, and RANKL is the key signaling molecule in this process.

### 5.3. Ferroptosis and OB

OB are one of the main cells involved in the bone reconstruction process, playing a dominant role in the synthesis, secretion, and mineralization of bone matrix, and the PI3K/Akt/mTOR pathway is an important pathway to regulate their bone formation function. In recent years, studies^[87–89]^ have found that iron accumulation is one of the important factors affecting osteoblastic bone formation ability; iron accumulation can produce ROS through the Fenton reaction and mitochondrial pathway; ROS can inhibit the PI3K/Akt pathway, leading to glycogen synthase kinase 3β (GSK-3β) activation; and it can further activate GSK-3β by inhibiting the Wnt signaling pathway. Runx2 is the main transcription factor of osteoblast differentiation, and GSK-3β phosphorylation can directly inhibit Runx2 on the one hand, and indirectly inhibit Runx2 through β-catenin degradation on the other hand, ultimately affecting osteoblast differentiation.^[90]^ In addition, the forkhead box-1 transcription factor, as a member of the FoxO family, can promote bone formation by inhibiting the oxidative stress response of OB, while the oxidative stress induced by iron accumulation can activate the PI3K/Akt pathway, inhibit the forkhead box-1 transcription factor, further aggravate the oxidative stress response, and inhibit the osteoblast bone formation ability.^[91]^ Xia D^[92]^ and Cen WJ^[93]^ and other studies have found that iron accumulation can downregulate the PI3K/AKT/FOX3a/DUSP14 signaling pathway, inhibit the growth and mineralization of osteoblasts, and induce G1 phase cell cycle arrest of osteoblast proliferation. Tian Q et al^[94]^ found through in vitro experiments that the RIPK1/RIPK3/MLKL signaling pathway is an important way for iron accumulation to promote osteoblast necrosis, and further found that PGAM5 and DRP1 as substrates of RIPK3 can maintain mitochondrial homeostasis, while PGAM5, upon activation by RIPK3, would be recruited to the mitochondrial membrane, activate DRP1 through dephosphorylation of serine 637 sites, and finally lead to the opening of mitochondrial membrane permeability conversion pores. Mitochondrial membrane potential loss and osteoblast death occur, and this process is affected by ROS. Xu G et al^[95]^ intervened in OB by ferric ammonium citrate and found that the levels of ROS, NADPH oxidase 4, and caspase-3 in osteoblasts increased significantly, and the activity of OB was significantly reduced, while the antioxidant N-acetyl-L-cysteine cleared ROS, which could reverse the death of osteoblasts induced by iron accumulation. In general, the process of ferroptosis in OB is regulated by the PI3K/AKT/FOXO3a/DUSP14 signaling pathway, the RIPK1/RIPK3/MLKL signaling pathway, and the Wnt signaling pathway, and ROS is a key target for iron accumulation-induced osteoblastic osteogenesis dysfunction.

## 6. Ferroptosis and orthopedic related diseases

### 6.1. Ferroptosis and SANFH

SANFH is a common clinical disease in orthopedics caused by the continuous use of glucocorticoids at high levels, and its incidence has increased year by year with the widespread use of glucocorticoids. SANFH is characterized by an imbalance of bone metabolism, intraosseous microthrombosis, vascular endothelial injury, deformation and collapse of the femoral head, and necrosis, often accompanied by symptoms such as pain and limited hip mobility.^[96]^ The pathogenesis of SANFH mainly includes apoptosis, cell pyroptosis, increased intraosseous pressure, vascular endothelial injury, and lipid metabolism disorders. However, recent studies have found that ferroptosis plays a necessary part in the occurrence and development of SANFH. Chen N et al^[97]^ collected 30 hormonal osteonecrosis samples and 10 nonhormonal osteonecrosis samples from the Gene Expression Comprehensive Database, identified differentially expressed genes (DEGs) between the 2 groups of samples, obtained ferroptosis-related genes from the FerrDb database, and then obtained ferroptosis-related DEGs at the crossover of ferroptosis-related genes and DEGs for KEGG and GO enrichment analysis. The genes and pathways associated with ferroptosis in the pathogenesis of hormonal osteonecrosis were verified, and the mechanism of ferroptosis in SANFH was further elucidated. Sun F et al^[98]^ found that dexamethasone can inhibit the expression of SLC7A11/GPX4, reduce the content of GSH in cells, increase malondialdehyde and ROS levels, as well as reduce mitochondrial volume and reduce mitochondrial crest, producing a series of obvious features of ferroptosis. Overexpression of SLC7A11 or use of a ferroptosis inhibitor (Fer-1) can improve dexamethasone-induced MC3T3 ferroptosis, proving that dexamethasone can activate p53/SLC7A11/the GPX4 pathway induces ferroptosis in MC3T3-E1 cells. Fang L et al^[99]^ after transfecting hormonal femoral head necrosis rats with SIRT6 adenovirus, by observing and comparing the microstructure of rat bones, alkaline phosphatase activity, expression levels of Runx2 and osteocalcin, and the ability of vascular endothelial cells to produce electricity, it was confirmed that glucocorticoids can increase intracellular Fe^2+^ and ROS levels, lead to ferroptosis, affect bone formation, and destroy microvascular endothelium, while SIRT6 can restore bone formation and angiogenesis by inhibiting ferroptosis. Thus preventing the occurrence of SANFH. The above studies provide a strong basis for the importance of cytosis in the pathogenesis of SANFH, and suggest that inhibition of ferroptosis will become a new and effective treatment for SANFH.

### 6.2. Ferroptosis and osteoporosis

Osteoporosis is a common clinical disease in orthopedics but also an “invisible killer” that threatens people’s health, and its prevalence is increasing year by year with the aging of the population. The main cause of osteoporosis is abnormal bone metabolism, in which the disruption of bone homeostasis is the key to the development of osteoporosis, which is often manifested as a decrease in osteoblast bone formation, an increase in osteoclastic bone resorption, and a weakening of osteogenic differentiation of BMSCs.^[100]^ In recent years, the molecular mechanisms of osteoporosis pathogenesis have been widely reported, and ferroptosis, as a new form of cell death, has become a hotspot of osteoporosis research.^[101]^ There is growing evidence that ferroptosis is an important factor in the onset and progression of osteoporosis.^[102]^ Iron overload can produce ROS through the Fenton reaction, and excess ROS activates multiple intracellular signaling pathways, enhances osteoclast bone resorption, inhibits osteoblast bone formation, and the bone reconstruction process is transformed into a pathological process of bone loss, leading to osteoporosis.^[83,103,104]^ Yang RZ et al^[36]^ found that the ROS in osteoblasts and osteocytes as well as the imbalance of redox reactions lead to an imbalance in bone metabolism, which plays an important part in glucocorticoid-induced osteoporosis pathogenesis. Ge W et al^[105]^ further experimented and found that the level of glycation end products increased in osteoporosis and confirmed that glycation end products can inhibit OB’s proliferation, differentiation, and mineralization by increasing the levels of malondialdehyde and free iron in osteoblasts, reducing GSH content, and inducing osteoblast ferroptosis, which in turn leads to osteoporosis. DNA methylation is closely related to ferroptosis and is one of the mechanisms of osteoporosis.^[106]^ As a classical pathway to promote bone formation, silence of JNK1-associated membrane protein inhibits the Wnt signaling pathway, whereas intragenic DNA methylation can inhibit osteogenic ability by inhibiting JNK1-associated membrane protein and Wnt signaling pathways.^[107]^ Hasegawa M et al^[108]^ found that mucin 1 can inhibit ferroptosis by binding to CD44 variants and enhancing the stability of SLC7A11, while silencing SLC7A11 promotes H3K9 methylation in mucin 1 promoters, affecting the production of GSH during ferroptosis. In addition, noncoding RNAs are also involved in iron accumulation mediating the pathogenesis of osteoporosis. Iron accumulation can lead to upregulation of lncRNA XIST and promote osteoblast death through the lncRNAXIST/miR-758-3p/caspase-3 axis, leading to osteoporosis,^[109]^ and regulation of the miR-203-3p/ZFPM2 axis by inhibiting lncRNA XIST can promote osteoblast proliferation and differentiation.^[110]^ Feng Y et al^[111]^ experimentally found that the viability of iron accumulation in mouse embryonic osteoblast precursor cells (MC3T3-E1) decreased, while the expression of miR-3074-5p in MC3T3-E1 cells increased significantly, and inhibition of miR-3074-5p could attenuate the level of MC3T3-E1 cell death mediated by iron accumulation. In conclusion, the above studies confirmed that the bone homeostasis imbalance mediated by iron accumulation is an important factor affecting the pathogenesis and progression of osteoporosis and that regulation of cellular ferroptosis can effectively prevent osteoporosis.

### 6.3. Ferroptosis and osteoarthritis

Osteoarthritis, as a chronic degenerative joint disease, is affected by a variety of factors (including age, obesity, trauma, metabolic syndrome, genetics, etc) and is characterized by degeneration of articular cartilage, sclerosis of subchondral bone, formation of bone redundancy, and inflammation of synovial disease, which seriously reduces the quality of life of patients. Due to the aggravation of population aging, the incidence of osteoarthritis is increasing.^[112]^ Therefore, the related research on osteoarthritis has received extensive attention from scholars, and with the deepening of the study of the pathogenesis of osteoarthritis, it has been found that osteoarthritis is closely related to iron accumulation.^[113,114]^ Iron accumulation induces lipid peroxidation of chondrocytes,^[115]^ OB and OC bone metabolism imbalance,^[116,117]^ macrophages,^[118]^ neutrophils,^[119]^ and fibroblasts function abnormally,^[120]^ which in turn leads to cartilage degeneration, subchondral bone destruction, and synovitis, accelerating the occurrence and development of osteoarthritis. In order to elucidate the direct effect of ferroptosis on the pathogenesis of osteoarthritis, Burton LH et al^[121]^ by injecting iron dextrose into guinea pigs with a low incidence of osteoarthritis, found that the guinea pigs had elevated iron content in the knee joint tissue, with mild irregular areas on the tibial surface, decreased proteoglycan content in cartilage, and decreased chondrocytes, which confirmed that there was a close relationship between iron accumulation and osteoarthritis. Yao X et al^[122]^ experimentally found that ferric ammonium citrate can promote the accumulation of ROS and lipid peroxides and the expression of ferroptosis-related proteins in chondrocytes, and that erastin, a ferroptosis inducer, is able to promote the expression of matrix metalloproteinase 13, inhibit the expression of type II collagen in chondrocytes, and further confirm that the antioxidant Fer-1 can reduce ferric ammouium citrate-induced cytotoxicity, reduce ROS and lipid peroxide levels, inhibits the expression of ferroptosis-related proteins, promote the expression of collagen II, and delay cartilage degradation and the process of osteoarthritis. Miao Y et al^[123]^ found that 55 patients with osteoarthritis had elevated levels of iron ions in the synovial fluid and significantly reduced levels of SLC3A2 and GPX4 in cartilage, and that down-regulation of GPX4 increased cartilage cell sensitivity to oxidative stress, up-regulated cartilage catabolism-related proteins, such as MMP3 and matrix metalloproteinase 13, and promoted the degradation of chondrocytes extracellular matrix through the MAPK/NF-κB pathway, while Fer-1 and deferoxamine can delay the process of osteoarthritis. It has been confirmed that ferroptosis does exist during the onset of osteoarthritis, and it is thought that inhibition of ferroptosis can be an effective treatment for osteoarthritis.

## 7. Conclusions and perspectives

BMSCs, OB, and OC are essential for bone shaping and reconstruction, and bone metabolism disorders and bone homeostasis imbalances mediated by abnormal function of BMSCs, OB, and OC play an irreplaceable role in bone and joint diseases, including SANFH, osteoporosis, and osteoarthritis. Therefore, the regulation of the activity and function of BMSCs, OB, and OC has received extensive attention from scholars. As a new form of programmed cell death, ferroptosis is involved in the occurrence and development of many diseases. Many studies have confirmed that ferroptosis can promote osteoclast bone resorption, inhibit osteoblast bone formation, affect bone formation and differentiation in BMSCs, disrupt bone homeostasis, further mediate the occurrence of the above related diseases, and accelerate their process. Therefore, there is good evidence that regulating cellular ferroptosis can be a proven treatment. This article reviews the occurrence and regulation mechanism of ferroptosis and the effects of ferroptosis on BMSCs, OB, and OC, clarifies the importance of targeted regulation of cellular ferroptosis to bone homeostasis, provides new ideas for bone metabolism research, and provides new directions for the clinical treatment of related orthopedic diseases. However, as a new form of cell death, ferroptosis and its mechanism of inducing bone homeostasis imbalance are complex, and there are still some mechanisms that mediate the occurrence of ferroptosis that are unclear, and some key targets in important signaling pathways need to be further explored, such as important targets for regulating oxidative stress and cell membrane repair and important pathways for regulating organelle function during ferroptosis. It is believed that in the near future, these problems will be overcome, and the way to regulate ferroptosis and prevent osteoarticular diseases will be more perfect.

## Author contributions

**Data curation:** Yongjie Zhang, Kangyi Hu.

**Funding acquisition:** Linzhong Cao.

**Resources:** Yongjie Zhang.

**Supervision:** Zhengya Shang, Xiaorui Yang, Linzhong Cao.

**Writing – original draft:** Yongjie Zhang.

**Writing – review & editing:** Yongjie Zhang, Linzhong Cao.
